# Supplementary figures and images for: Lactate and histone H3K18 lactylation are associated with metabolic control of gene expression in the retina
Source: PLoS Genet. 2026 Apr 8;22(4):e1012100. doi: 10.1371/journal.pgen.1012100 (PMC13095125; doi:10.1371/journal.pgen.1012100)

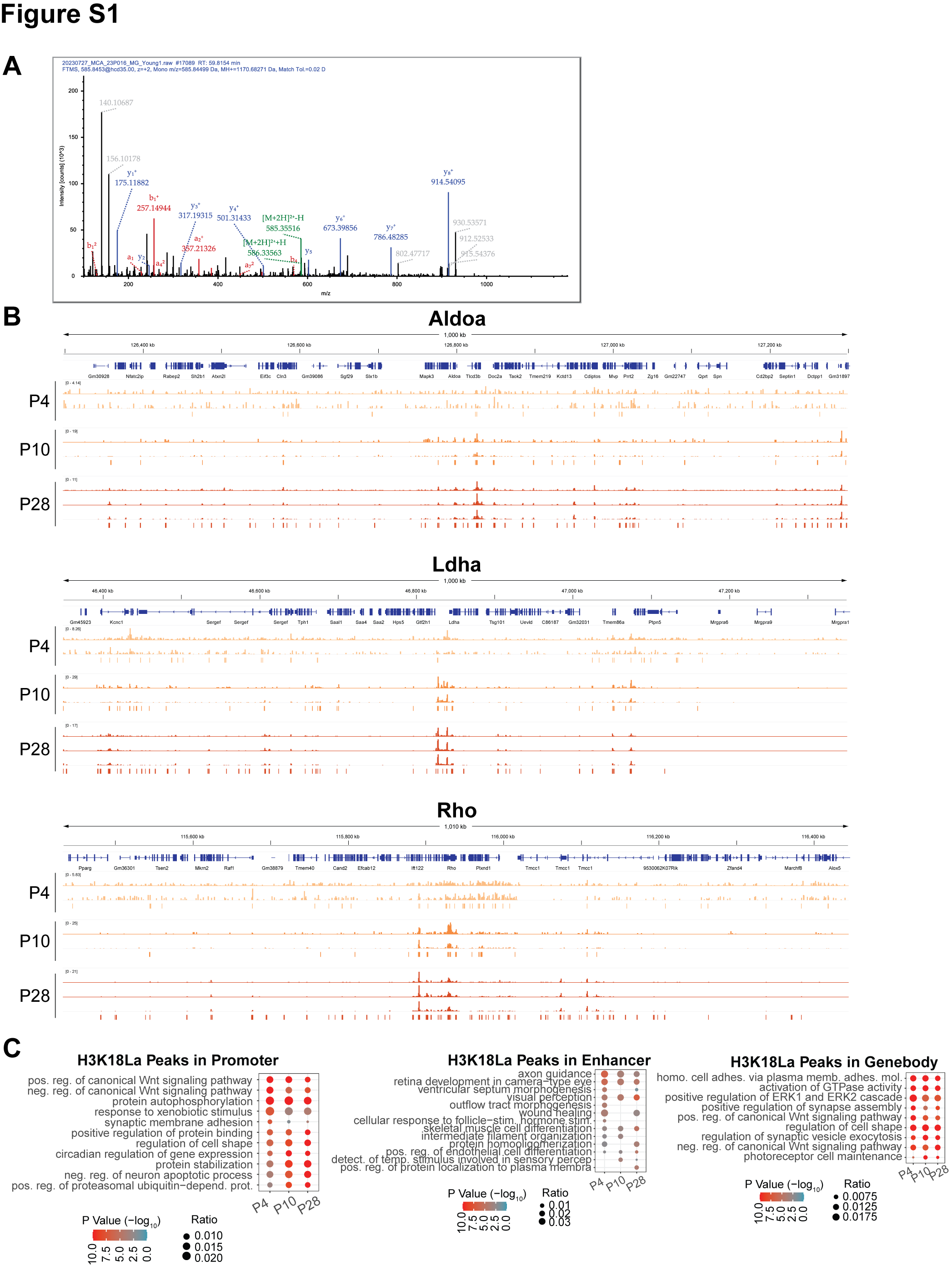

Supplement: S1 Fig — (A) Annotated HCD Spectrum of Histone H3.3 peptide R.KQLATKAAR.K modified with 1 × Propionyl [N-Term]; 1 × Propionyl [K6]; 1 × lactylation [K]; 1 × Acrolein [K1]. Peaks in blue are fragment ions containing the C-terminus (y-ions) and peaks in red are fragment ions containing the N-terminus (b-ions). (B) Genomic histogram traces for 1 megabase regions of H3K18La sample replicated at each timepoint for genes involved in glycolysis (Aldoa and Ldha) and the phototransduction cascade in rod photoreceptors (Rho). The histogram traces are group scaled for each individual timepoint. The bars under each timepoint histogram represent consensus peaks. (C) GO Biological Process gene sets enriched for genes containing H3K18La in each timepoint in different defined gene regions. (TIF) [file pgen.1012100.s001.tif]

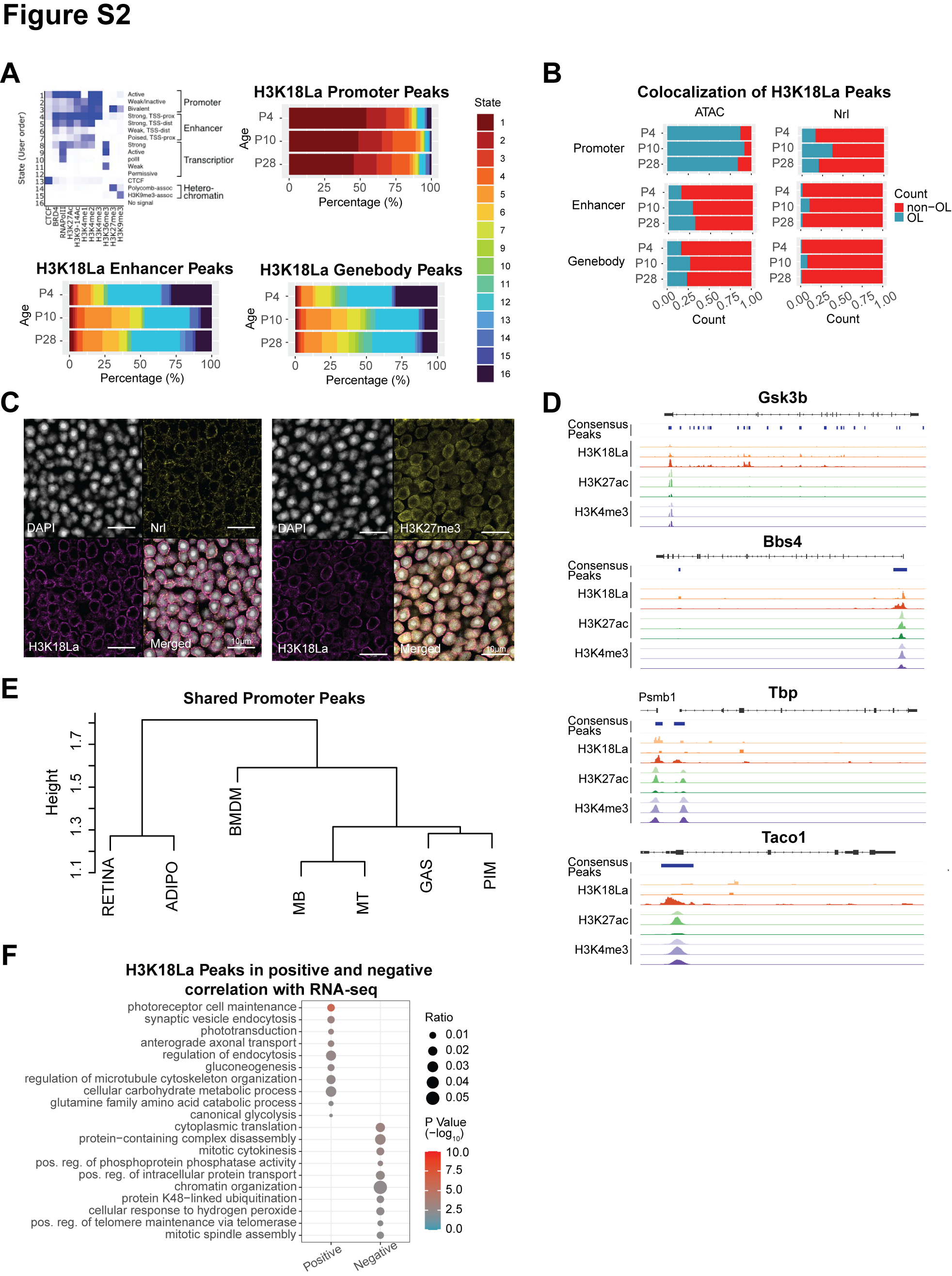

Supplement: S2 Fig — (A) H3K18La peaks are overlapped with 16 chromatin states defined by ChromHMM from retina ChIP-seq data [3] for each defined gene region and timepoint. (B) Colocalization of H3K18La peaks with H3K27Ac [3] bound regions. (C) Confocal immunofluorescent images showing colocalization of NRL and H3K27me3 with H3K18La in nuclear periphery of photoceptor cells. Scale bars, 10 μm. (D) Genomic histogram traces of histone marks during development for representative selected genes found in Fig 4H. H3K18La marks at P4, P10, and P28 (Red), H3K27Ac [3] at P3, P10, P21 (Green), and H3K4me3 at P3, P10, and P21 (Purple). (E) Hierarchical clustering of shared H3K18La promoter peaks between retina and other tissues [37]. (F) GO Biological Process gene sets enriched for protein coding genes containing H3K18La in the promoter which are positively or negatively correlated with RNA-seq expression during development. Abbreviations: GAS, Gastrocnemius; MT, Post-mitotic end-state myotubes; PIM, Post- ischemia macrophages; MB, Myoblasts; ADIPO, Adipose tissues; BMDM, Bone marrow-derived macrophages. (TIF) [file pgen.1012100.s002.tif]

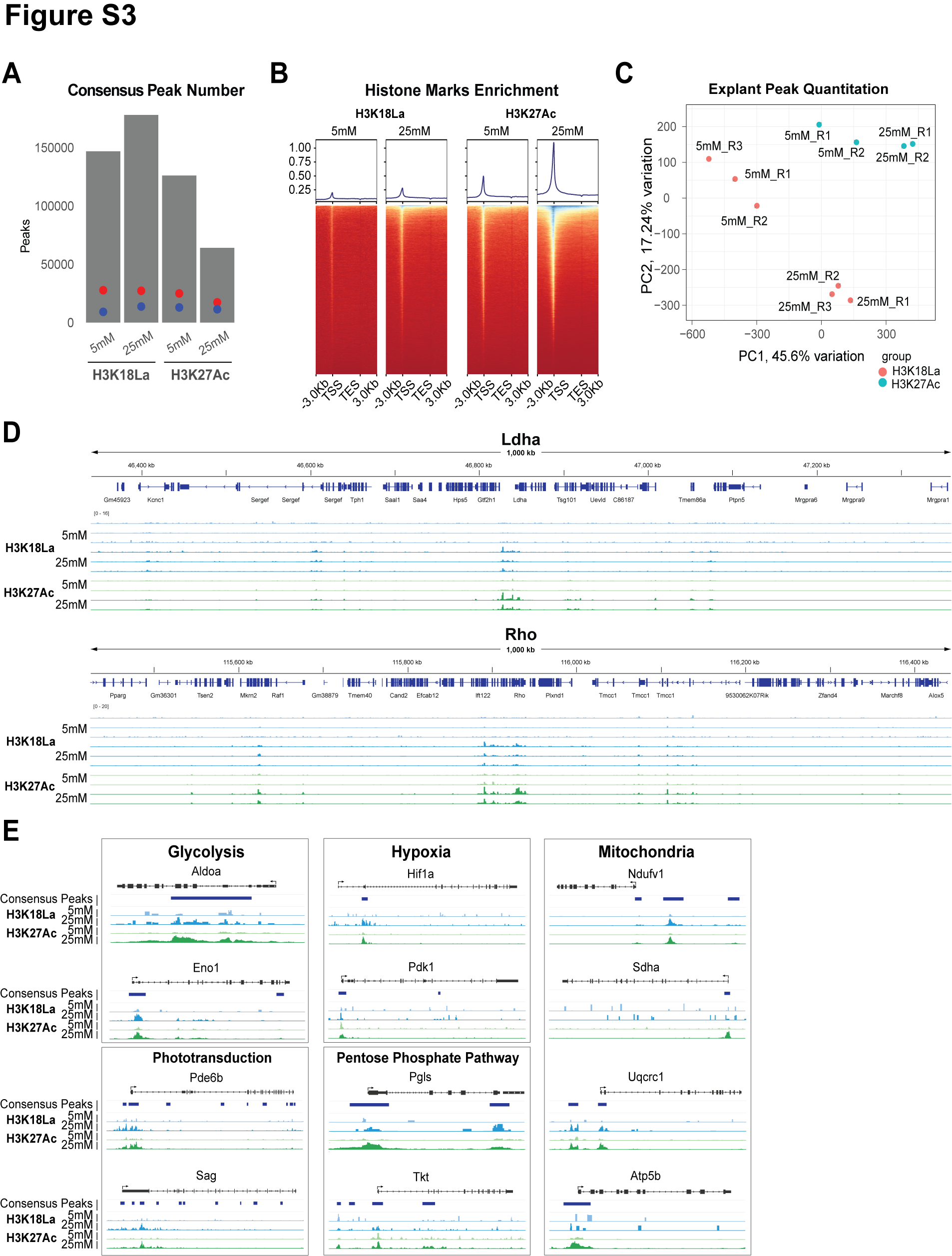

Supplement: S3 Fig — The number of consensus peaks passing a 1x10^-6^ FDR for each replicate per explant glucose concentration and histone mark. The red dot indicates the number of genes containing a peak, whereas the blue dot represents the number of genes containing a peak in the proximal promoter. (A) Heatmap of H3K18La-bound and H3K27Ac-bound peak signal enrichment relative to distance from TSS/TES for all genes and their flanking 3 kb region. (B) Principal component analysis of quantitative peak binding for all H3K18La and H3K27Ac samples. (D) Genomic histogram traces for 1 megabase regions of H3K18La and H3K27Ac sample replicates at each glucose conditions (5 mM and 25 mM) for genes involved in glycolysis (Ldha) and the phototransduction cascade in rod photoreceptors (Rho). The histogram traces are group scaled for each individual timepoint. The bars under each timepoint histogram represent consensus peaks. (E) Genomic histogram traces of H3K18La and H3K27Ac in explants for representative genes of several affected pathways observed. The histogram traces are group normalized for each gene. The bars under each gene track represent consensus peaks. Abbreviations: FDR, false discovery rate; PCA, principal component analysis; PC1, principal component 1; PC2, principal component 2; DB, Differentially bound. (TIF) [file pgen.1012100.s003.tif]

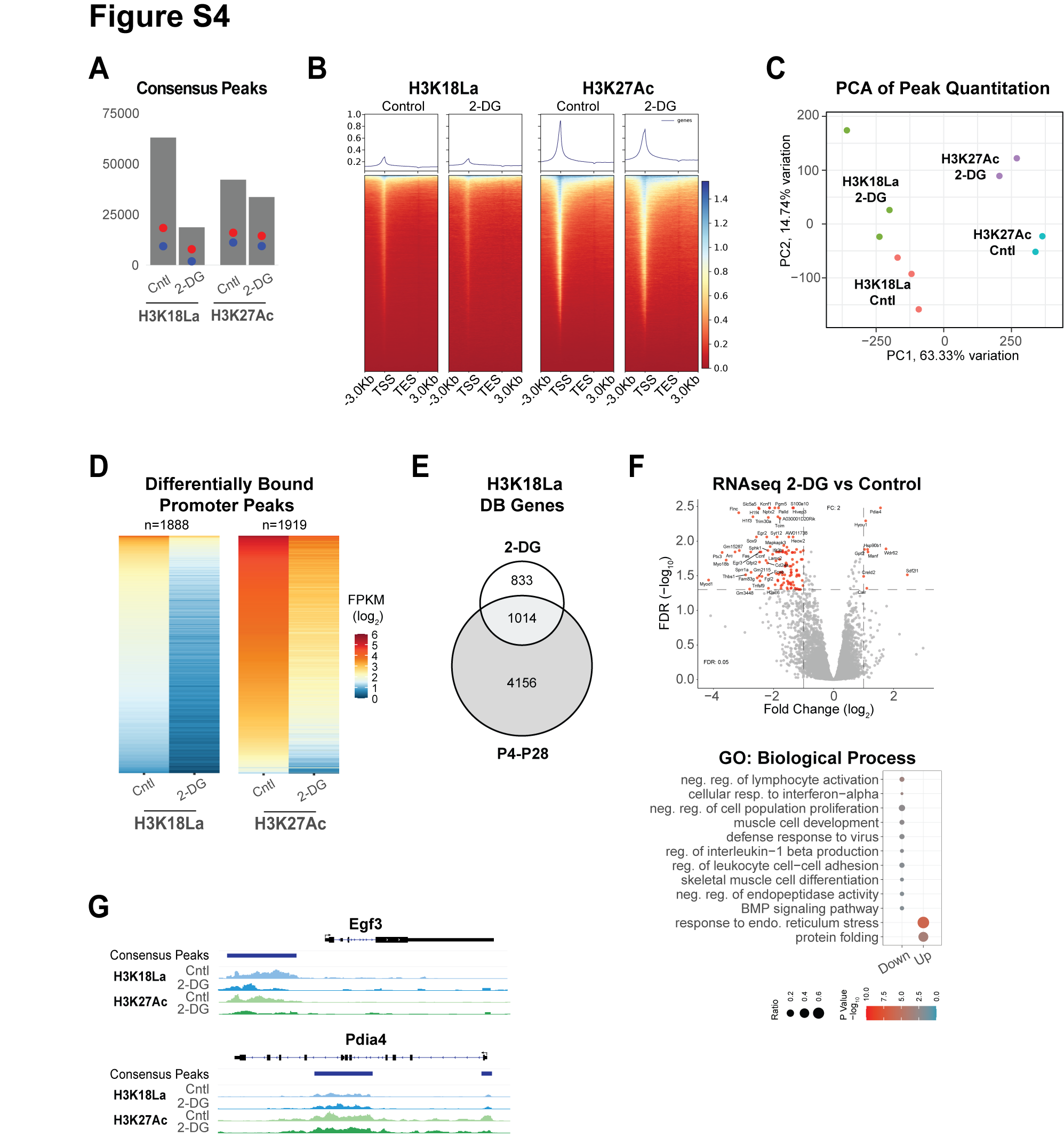

Supplement: S4 Fig — (A) The number of consensus peaks passing a 1x10^-6^ FDR for each replicate per explant 2-DG concentration and histone marks. The red dot indicates the number of genes containing a peak, whereas the blue dot represents the number of genes containing a peak in the proximal promoter. (B) Heatmap of H3K18La and H3K27Ac-bound peak signal enrichment for all genes and their flanking 3kb region under control (no inhibitor) and 2-DG (20 mM, 48 hour) conditions. (C) PCA of CUT&Tag quantitative peak binding separates controls from 20 mM 2-DG-treated retinal explants for H3K18La and H3K27Ac. (D) Differentially bound promoter peaks for H3K18La (Left) and H3K27Ac (Right) for 2-DG versus control in retinal explants. n denotes the number of peaks. (E) Number of genes containing differential bound promoter peaks for H3K18La from 2-DG versus control in retinal explants compared to retinal development (P4-P28). (F) RNA-seq expression analysis for retinal explants (2-DG versus contro). Horizontal line: FDR = 0.05; vertical lines: |log2FC| = 1 (fold-change = 2). Red dots are significant differentially expressed genes. Gene Ontology Biological Process analysis of genes differentially expressed in RNA-seq following 2-DG treatment versus control. (G) Genomic histogram traces of explant histone marks for representative genes of several affected pathways observed. The histogram traces are group normalized for each gene. The bars under each gene track represent consensus peaks. Abbreviations: TSS, transcription start site; TES, transcription end site; PCA, principal component analysis; PC1, principal component 1; PC2, principal component 2; FDR, false discovery rate; logFC, log fold-change. (TIF) [file pgen.1012100.s004.tif]

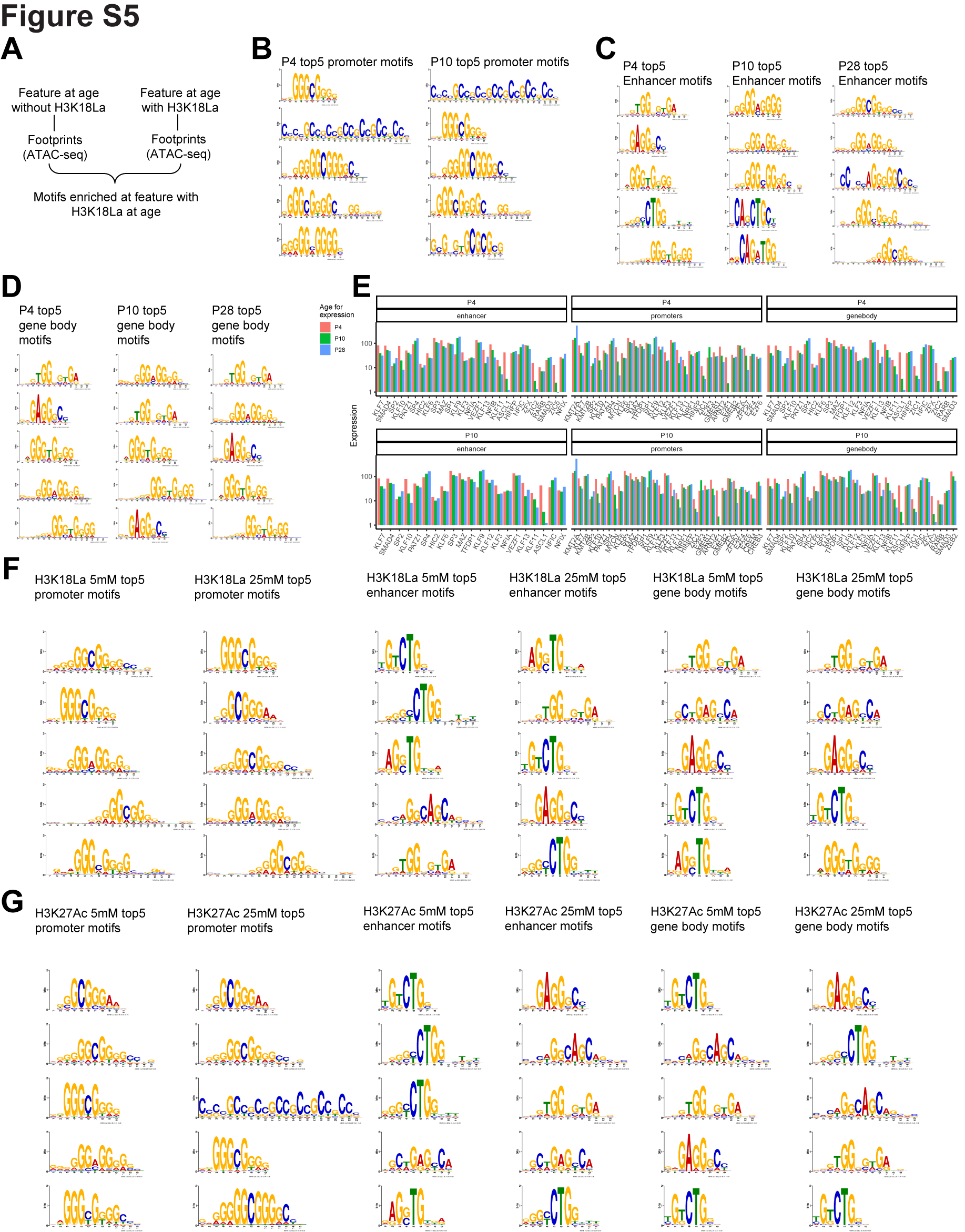

Supplement: S5 Fig — (A) Accessible motifs enrichment analysis pipeline. (B) Top 5 accessible motifs enriched at H3K18La promoters at P4 (left) and P10 (right). (C-D) Top 5 accessible motifs enriched at H3K18La enhancers (C) and gene bodies (D) at P4 (left) and P10 (center) and P28 (right). (E) Expression levels of expressed TF from the 100 top enhancers, promoters or gene bodies motifs at P4 and P10. (F-G) Top 5 accessible motifs enriched at H3K18La (F) or H3K27Ac (G) promoters, enhancers and gene bodies. (TIF) [file pgen.1012100.s005.tif]

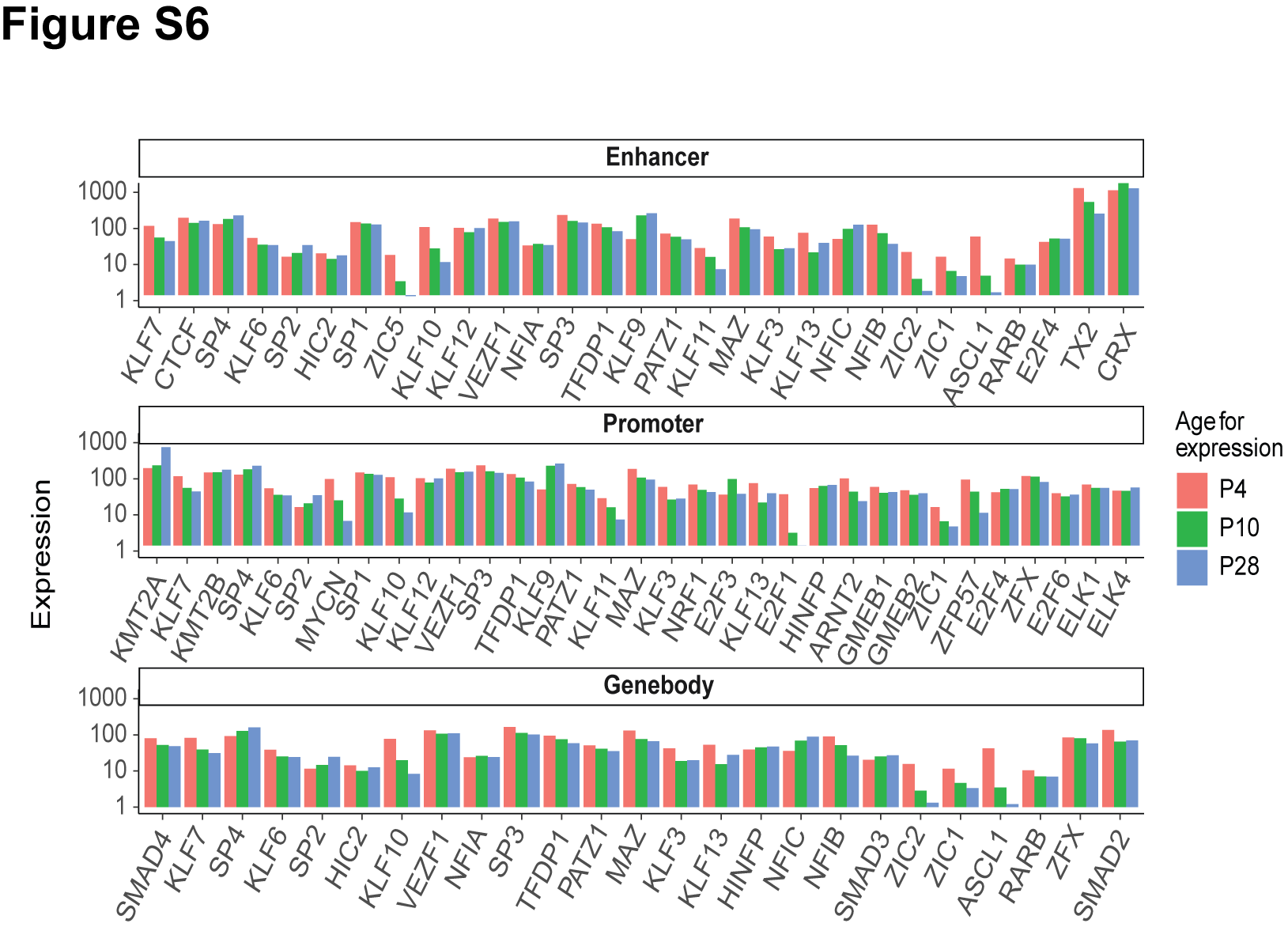

Supplement: S6 Fig — Expression levels of expressed TF from the 100 top enhancers, promoters or gene bodies motifs at P28. (TIF) [file pgen.1012100.s006.tif]
