# Supplementary material for: Lactate and histone H3K18 lactylation are associated with metabolic control of gene expression in the retina
Source: PLoS Genet. 2026 Apr 8;22(4):e1012100. doi: 10.1371/journal.pgen.1012100 (PMC13095125; doi:10.1371/journal.pgen.1012100)
Supplement: Attachment — Submitted filename: Review of Gaur et al. for Plos Genetics, 2026.docx [file pgen.1012100.s018.docx]

Gaur et al., 2026 Plos Genetics - Manuscript Review

**Summary and Overall Opinion**

Gaur et al. presents data which suggests or shows:

(A) an increase in glycolysis over development (this has been covered in previous manuscripts as well, see PMID: 13618528
(B) photoreceptor-specific increases in H3K18La over the course of development
(C) Strong H3K18La and pan-Kla labeling in the inner retina
(D) A high-density of H3K18La near the TSS (though total amounts summed across the gene body are greater)
(E) Increases in H3K18La with retina development
(F) An association between glycolytic flux and H3K18La, which is stronger than the association between glycolysis and H3K27Ac (though from the data, the association between glycolysis and pan-Kla labeling seems strongest)
(G) Enrichment of H3K18La on GC-containing motifs

There are some analytical elements of the genomics data that I am not qualified to evaluate, but from the above points alone I think there is plenty of novel and useful information that merits publication. I do not fully agree with a couple author interpretations of some of the data (for example, co-association in the immunofluorescent images or correspondence between H3K18La changes and transcript changes), so it would be nice to see the data quantified in a way that supports statements of association (correlation coefficients for example). There are some elements of the data which I am not sure add to the conclusions of this study (PCA plots, peak counts), so their importance should be emphasized more clearly.

The only major changes I would suggest include an assessment of cell death with 2-deoxy-glucose, and an assessment of final [glucose] in medium in which retinas had been incubated, for all ex vivo incubation experiments. These additional data will provide essential context for the experiments that the authors present.

Overall, this will be new and useful data to the scientific community which shows associations between retina development, metabolism, and metabolite-dependent epigenetic marks.

**Specific concerns and comments**

- Page 5, *“The rod and cone photoreceptors account for almost 75% of the cells in most mammalian retina”* – My understanding was that it’s closer to ~60%, which appears to be the case in mouse retinas (PMID: 9786999). I cannot find photoreceptor densities or proportions in the cited review (reference #20). I could be missing it, but please double check to make sure that you are citing the paper you intended, and that the reference indeed has this information.
- Page 8, “*To test this hypothesis, we first performed high-resolution mass spectrometry analysis of histones from the P28 mouse retina. Lysine lactylation (KLa) is abundantly present across histone proteins (Fig 2B and S1A Fig and S1 Table)”* – The language of this sentence suggests that you have data showing KLa on many distinct histone lysines. The cited figures show (2B) a diagram of potential Kla sites, (S1A) a single H3.3 peptide with one Kla, and (table S1) an excel sheet with many peptides, where none of the annotations specify that they contain KLa. What might be an informative alternative to this table is a version of Fig2B where you have an amino acid sequence and at each lysine residue, a histogram which indicates the proportion of that peptide that is lactylated at that lysine. This is only a suggested alternative to 2B, though it is crucial that you communicate more effectively what is data, and what that data expresses.
- Page 8 – Of the many possibly lysine lactylation sites, only H3K18La and H4K12La are subjected to immunoblot and fluorescent analysis. It would improve the flow of the manuscript if greater context was given for focusing on the developmental dynamics of these residues. One reason could be that these lysines are highly lactylated. Another might be the presence of previous literature suggesting that one of these sites is developmentally significant.
- Page 14 – The 2-deoxyglucose experiments are an important element of this study, but the authors are missing a key piece of information. How many cells have died when glucose cannot reach the retina? Are we seeing differential levels of lysince acylation due to the fact that fewer cells can tolerate long-term inhibition of glycolysis? This is my instinctive interpretation of the experiment. **It will be crucial for the authors to measure retinal cell death under these conditions.** If there is cell death, would a cytotoxic agent that does not alter glucose but causes the same amount of cell death also change histone profiles comparably? Without a cell death measurement, the interpretation of this experiment could be wrong.
- Page 23/24, *“Limitations of the present study” –* Measurements of O_2_ consumption by ex vivo retina tissue appear to be limited by the diffusion of O_2_ into the center of the tissue (PMID: **41138202). You note that explant cultures do not recapitulate RPE-retina glucose shuttle. I suggest modifying the statement to include that OCR/ECAR measurements from ex vivo retina cultures could be affected by limited** O_2_ supply.
- Page 20, *“Discovery of acyl marks on histones, such as propionyl…”* – A minor language edit, I suggest ending the sentence in “lactyl-lysine”. Otherwise readers might be led to think that the modifications are lactyl-CoA-lysine all together.
- Page 24/Figure 5, *“Retina Isolation and Explant Culture”*, The protocol is a bit confusing. The schematic in A suggests that the only incubation period is one 48-hour long incubation. The methods section suggests that a 1-hour long incubation in KRB follows the 48-hour incubation. Please clarify which is accurate, as these different experimental setups will almost certainly lead to different outcomes. If the latter is accurate, it would be best to represent this in the text and schematic. It would also be important to indicate the source or composition of the KRB, as some commercial formulations are made with glucose, and some are not. Is there any particular purpose to the 48-hour holding period? If so it might be worth mentioning the motivation for this experimental setup. Notably, if retinas are supplied with ~1-2 mL of 5mM glucose, they can entirely deplete this glucose in <24 hours. Some of these retinas may be starving for glucose under the conditions outlined here, including what might be considered the 5 mM “control”. **For the sake of understanding the state of the retina at the time of the assay, I strongly recommend measuring glucose concentration in medium after the 48-hour incubation period.**
- Page 28, “*with formulars described previously [63].” – spelling typo*
- Page 28/Figure 5B, 5D – The lactate assay protocol in the methods seems to be for tissue. The figure panels specify that secreted lactate is being measured. Please clarify which. Please clarify if the medium or tissue used for lactate estimation is from 48-hour neurobasal medium or 1 hour KRB.
- Figure 2A – I understand that you should not need to include every intermediate step in this figure panel, but I do think that one crucial step is missing, the conversion of lactate to lactyl-CoA prior to lysine acylation by ACSS2 (PMID: **39561764)**.
- Figure 3F and S2C – the level of colocalization with H3K18La and NRL seems stronger than it is portrayed in the text, and overlap between H3K27me3 also seems strong, if only because signal for H3K27me3 is everywhere. Could you use an image analysis tool (for example from imageJ) to better support or refute these claims related to colocalization on pages 10 and 11: “*Confocal microscopy using specific antibodies validates partial colocalization of H3K27Ac and H3K18La signals in the euchromatin region at the periphery of photoreceptor nuclei (Fig 3F). Little or no colocalization is detected between H3K18La and H3K27me3 (a mark associated with reduced gene expression) or NRL immunolabeling (S2C Fig). This suggests that H3K18La is enriched at euchromatic promoters in photoreceptors where it overlaps with H3K27Ac, while remaining distinct from the H3K27me3, a repressive mark.)”.*
- Figure 3J - I don’t quite understand this panel. It is supposed to represent H3K18La co-occupancy at promotors. Co-occupancy with what other factor? The y-axis says “intersection”, but I am not sure what thing that is intersecting is being counted. Some of the bar graphs are for only one tissue (retina, BMDM, ADIPO, and MB), others are for more than one, but there is no clear explanation of why particular tissue pairings are chosen to be shown here.
- Figure 4F shows several different temporal patterns where transcript expression can increase before, during, or after increases in H3K18La. I am not a genomics expert, but does this not devalue the claim that H3K18La causes an increase in transcription? It seems to me that this panel shows evidence that H3K18La does not exert a strong level of control over transcription. Of course I understand that each of these genes will be subjected to transcriptional control by an abundance of other factors in addition to H3K18La, but there should
- Figure 5C – a label is missing on the right-most panel. I assume it is H3K27Ac.
- Figure 6G – a box around the different pathways (glycolysis vs. PPP vs. mitochondrial) will help to better indicate which gene is associated with which pathway.
- Figures 6 and 7 - The manuscript could be simplified if the data in figures 6 and 7 were combined. The goal of these figures are to interrogate the epigenetic responses to increases (fig 6) and decreases (fig 7) in glucose metabolism. These modulations, place on a single scale, could yield a more comprehensive glycolytic dose-response effect.
- Figure 7G – This figure shows a rather poor correlation between changes in transcript levels and changes in H3K27Ac or H3K18La. Does an integrated measure of both H3K27Ac and H3K18La cut-and-tag datasets improve the ability of these histone marks to predict transcript levels?
- I am not a genomics expert, so I am not sure what information is gained from the PCA plots and the # of consensus peaks which isn’t supported by other elements of the manuscript. With the goal of simplifying the manuscript, I suggest moving these data to the supplemental text.
- Discussion – This is an opinion, but this section contains a lot of information typically destined for the introduction. I recommend removing the first ~8 lines of text in the first paragraph, and there are many examples of text which does not add to the section which should be focused on the interpretation of your findings rather than a re-introduction to the field.
- Page 21 line 3. The sentence which begins on this line is totally unsupported by the reference (ref 51), which neither mentions the AARS1 gene or lactyl-CoA. A Pubmed search of “AARS1 and Lactyl-CoA” gives no results, though a google search supports the statement (PMID: **38653238**, 38512451). Perhaps this is an error with the reference manager (we’ve all dealt with these), but this means the authors should comb through and make sure all references are properly attributed.
- Methods – there are catalog numbers missing from many items. To ensure reproducibility, please include these in the final submission of this manuscript.
