# Supplementary material for: Lactate and histone H3K18 lactylation are associated with metabolic control of gene expression in the retina
Source: PLoS Genet. 2026 Apr 8;22(4):e1012100. doi: 10.1371/journal.pgen.1012100 (PMC13095125; doi:10.1371/journal.pgen.1012100)
Supplement: Attachment — Submitted filename: Gaur PLoS Genet Response_Reviewers_02252026.docx [file pgen.1012100.s020.docx]

**Response to the Reviewers’ Comments**

**Reviewer #1:**

Gaur et al. presents data which suggests or shows:

(A) an increase in glycolysis over development (this has been covered in previous manuscripts as well, see PMID: 13618528
(B) photoreceptor-specific increases in H3K18La over the course of development
(C) Strong H3K18La and pan-Kla labeling in the inner retina
(D) A high-density of H3K18La near the TSS (though total amounts summed across the gene body are greater)
(E) Increases in H3K18La with retina development
(F) An association between glycolytic flux and H3K18La, which is stronger than the association between glycolysis and H3K27Ac (though from the data, the association between glycolysis and pan-Kla labeling seems strongest)
(G) Enrichment of H3K18La on GC-containing motifs

There are some analytical elements of the genomics data that I am not qualified to evaluate, but from the above points alone I think there is plenty of novel and useful information that merits publication. I do not fully agree with a couple author interpretations of some of the data (for example, co-association in the immunofluorescent images or correspondence between H3K18La changes and transcript changes), so it would be nice to see the data quantified in a way that supports statements of association (correlation coefficients for example). There are some elements of the data which I am not sure add to the conclusions of this study (PCA plots, peak counts), so their importance should be emphasized more clearly.

The only major changes I would suggest include an assessment of cell death with 2-deoxy-glucose, and an assessment of final [glucose] in medium in which retinas had been incubated, for all ex vivo incubation experiments. These additional data will provide essential context for the experiments that the authors present.

Overall, this will be new and useful data to the scientific community which shows associations between retina development, metabolism, and metabolite-dependent epigenetic marks.

*Response: We thank the reviewer for their positive assessment that our study provides novel and useful information meriting publication. We also appreciate the constructive suggestions to further strengthen our study.*

*We agree that quantification of IHC data will strengthen our interpretations. We have now performed the colocalization analysis, and results are being presented in the revised text. To focus on the biological question whether two marks occupy the same subnuclear areas, we report the voxel-based overlap within a defined Region of interest (%ROI colocalized) after objective Costes thresholding. This metric is more informative because H3K27me3 exhibits comparatively diffuse nuclear staining with a broad intensity distribution.*

*We have also revised the organization of PCA and consensus peak number analyses. Specifically, we have moved the PCA plots and consensus peak number plots associated with the retinal explant experiments to the Supplementary Information to streamline the main text. However, we have retained the consensus peak analysis for the developmental time points in figure 3 and the PCA plots in figure 4 (CUT&Tag and RNA-seq during development). We believe these analyses are essential in the developmental context, as they demonstrate the high reproducibility of biological replicates and establish that the observed chromatin and transcriptional changes represent consistent, global shifts rather than locus-specific effects. We have also revised the figure legends to more clearly explain the purpose and interpretation of these analyses.*

*We also appreciate reviewer’s suggestion and acknowledge that prolonged inhibition of glycolysis can be cytotoxic to retinal cells. However, our study was designed to capture the epigenetic response to metabolic perturbation. Future studies would be required to correlate the timeline of these epigenetic modifications with the progression of cell death. We do not actually see any visible sign of cell death, but we did not perform specific assays for cell death (e.g., TUNEL). Previous studies, nonetheless, have shown that* *2-DG does not induce cell death in the effective range established by multiple groups, i.e., 5mM to 20mM for 48hrs (see https://doi.org/10.7554/eLife.100604.3). We have now clarified that we are in the effective range by adding the following sentence in the methods: “**different concentration of 2-DG (10 mM and 20 mM for immunoblots and 20 mM for CUT&Tag, range that is typically used in cell culture studies)”.*

**Specific concerns and comments**

- Page 5, *“**The rod and cone photoreceptors account for almost 75% of the cells in most mammalian retina”* – My understanding was that it’s closer to ~60%, which appears to be the case in mouse retinas (PMID: 9786999). I cannot find photoreceptor densities or proportions in the cited review (reference #20). I could be missing it, but please double check to make sure that you are citing the paper you intended, and that the reference indeed has this information.

*Response: We sincerely thank the reviewer to provide a more accurate and appropriate reference. The reviewer is correct; our citation of Lamb (2013) was for the broader context of photoreceptor biology and was inappropriately used to support a specific quantitative claim.*

*Notably, in the Jeon et al. 1998 reference, provided by the reviewer, the stated proportion of rods in the mouse retina is consistent with established quantitative analyses of retinal cell composition, and the* ***rods constitute 75–80% of total retinal cells (see Figure 7 in Jeon et al.****). Our statement is therefore accurate and supported by the published literature. To avoid any ambiguity, we have now added the citation with the more precise reference suggested by the reviewer (Jeon et al., 1998). We apologize for the oversight*.

- Page 8, “*To test this hypothesis, we first performed high-resolution mass spectrometry analysis of histones from the P28 mouse retina. Lysine lactylation (KLa) is abundantly present across histone proteins (Fig 2B and S1A Fig and S1 Table)”* – The language of this sentence suggests that you have data showing KLa on many distinct histone lysines. The cited figures show (2B) a diagram of potential Kla sites, (S1A) a single H3.3 peptide with one Kla, and (table S1) an excel sheet with many peptides, where none of the annotations specify that they contain KLa. What might be an informative alternative to this table is a version of Fig2B where you have an amino acid sequence and at each lysine residue, a histogram which indicates the proportion of that peptide that is lactylated at that lysine. This is only a suggested alternative to 2B, though it is crucial that you communicate more effectively what is data, and what that data expresses.

*Response: We appreciate the reviewer’s careful reading of Fig. 2B, which represents a data-driven summary of the number of distinct lysine lactylation sites identified on histone proteins by high-resolution mass spectrometry. We have revised the text to avoid implying quantitative site occupancy and now state that multiple lysine lactylation sites were identified across histones. We have also clarified the Fig. 2B legend to* *reflect the number of unique lactylated lysines identified per histone. We have updated the Methods text “**In our mass spectrometry search parameters,* *lysine lactylation was identified as a variable modification with a mass shift of +72.021 Da, which is annotated by the search engine as 'Carboxyethyl' (Table S1). For clarity and consistency with established biological nomenclature, all 'Carboxyethyl' annotations are referred to as 'Lactylation' (KLa) throughout the manuscript and figures”.*

- Page 8 – Of the many possibly lysine lactylation sites, only H3K18La and H4K12La are subjected to immunoblot and fluorescent analysis. It would improve the flow of the manuscript if greater context was given for focusing on the developmental dynamics of these residues. One reason could be that these lysines are highly lactylated. Another might be the presence of previous literature suggesting that one of these sites is developmentally significant.

*Response: We thank the reviewer for this thoughtful suggestion. Among the reported lysine lactylation sites, H3K18La and H4K12La are the most extensively characterized in prior studies. Previous reports have demonstrated that H3K18La is enriched at active regulatory regions, particularly tissue-specific enhancers, and is associated with gene activation in defined biological contexts. And, H4K12La is shown to mediate a positive feedback loop, exacerbating microglial dysfunction in Alzheimer disease with change in glucose metabolism. We have clarified the context of using these antibodies with the statement, “We then performed immunoblot analysis using antibodies against H3K18La and H4K12La since these two histone lysine lactylation sites are reported to link glucose metabolism to epigenetic regulation [**7**,* *36**].”*

- Page 14 – The 2-deoxyglucose experiments are an important element of this study, but the authors are missing a key piece of information. How many cells have died when glucose cannot reach the retina? Are we seeing differential levels of lysince acylation due to the fact that fewer cells can tolerate long-term inhibition of glycolysis? This is my instinctive interpretation of the experiment. **It will be crucial for the authors to measure retinal cell death under these conditions.** If there is cell death, would a cytotoxic agent that does not alter glucose but causes the same amount of cell death also change histone profiles comparably? Without a cell death measurement, the interpretation of this experiment could be wrong.

*Response: We thank the reviewer for clarifying their concerns about the inter-pretation of our 2-DG experiment. We agree that without a measure of cell death, we cannot definitively separate the specific molecular consequences of glycolytic inhibition from the general, non-specific effects of cytotoxicity. We have therefore revised our interpretation and discussion in the manuscript to incorporate this uncertainty. And the data presented in our manuscript should be interpreted as a snapshot of the epigenetic state of a retina undergoing metabolically induced stress through 2-DG.*

- Page 23/24, *“**Limitations of the present study” –* Measurements of O_2_ consumption by ex vivo retina tissue appear to be limited by the diffusion of O_2_ into the center of the tissue (PMID: **41138202). You note that explant cultures do not recapitulate RPE-retina glucose shuttle. I suggest modifying the statement to include that OCR/ECAR measurements from ex vivo retina cultures could be affected by limited** O_2_ supply.

*Response: We appreciate the reviewer for this important technical point. As suggested, we have revised the "Limitations of the present study" section and added the suggested line.*

- Page 20, *“**Discovery of acyl marks on histones, such as propionyl…”* – A minor language edit, I suggest ending the sentence in “lactyl-lysine”. Otherwise readers might be led to think that the modifications are lactyl-CoA-lysine all together.

*Response: We thank the reviewer for this edit. The reviewer is correct that 'acyl-CoA' refers to the donor molecule. We have revised the sentence (previously on page 20, now page 29) to now read: “The discovery of acyl marks on histones, such as propionyl, butyryl, crotonyl, and the more recent lactyl-lysine, has greatly enhanced our knowledge of functional diversity of histone modifications [**5**].”*

- Page 24/Figure 5, *“Retina Isolation and Explant Culture”*, The protocol is a bit confusing. The schematic in A suggests that the only incubation period is one 48-hour long incubation. The methods section suggests that a 1-hour long incubation in KRB follows the 48-hour incubation. Please clarify which is accurate, as these different experimental setups will almost certainly lead to different outcomes. If the latter is accurate, it would be best to represent this in the text and schematic. It would also be important to indicate the source or composition of the KRB, as some commercial formulations are made with glucose, and some are not. Is there any particular purpose to the 48-hour holding period? If so it might be worth mentioning the motivation for this experimental setup. Notably, if retinas are supplied with ~1-2 mL of 5mM glucose, they can entirely deplete this glucose in <24 hours. Some of these retinas may be starving for glucose under the conditions outlined here, including what might be considered the 5 mM “control”. **For the sake of understanding the state of the retina at the time of the assay, I strongly recommend** **measuring glucose concentration in medium after the 48-hour incubation period.**

*Response: We thank the reviewer for this insightful critique of our experimental presentation. First, we apologize for the confusion caused by our schematic (Fig 5A). The reviewer is correct that the schematic was incomplete. We have now added that in the schematic in figure 5A and in the methods text .*

*With respect to potential glucose depletion during the 48-hour incubation, we performed daily medium replacement (50% with pre-warmed conditioned medium), which helps maintain nutrient availability and tissue viability. We apologize for not clearly stating this in the method text, we have now modified the method with the line “with daily medium replacement of 50% media with pre-warmed conditioned medium”. We agree that glucose consumption is an important consideration in metabolically active tissues such as the retina. We did not directly measure residual glucose levels in the medium at the end of the incubation period, we followed strictly what has been published for retinal explants from different groups that demonstrates retinal explants remain viable and structurally preserved during multi-day culture. Also, we believe that time course experiments with labelled glucose should be done to dissect the direct correlation between glucose metabolic changes and histone lactylation as we also pointed out in our discussion.*

- Page 28, “*with formulars described previously [63].” – spelling typo*

*Response: We thank the reviewer for catching this spelling error. The typo is corrected from 'formulars' to 'formulas' in the revised manuscript.*

- Page 28/Figure 5B, 5D – The lactate assay protocol in the methods seems to be for tissue. The figure panels specify that secreted lactate is being measured. Please clarify which. Please clarify if the medium or tissue used for lactate estimation is from 48-hour neurobasal medium or 1 hour KRB.

*Response**: We thank the reviewer for pointing out this ambiguity. In the retinal explant experiments (figure 5),* *lactate levels were measured in the conditioned medium, not in retinal tissue. Specifically, we quantified lactate released into the medium during the 1-hour KRB incubation period. In contrast, for the developmental time-course experiments shown in figure 1F, lactate levels were measured directly from retinal tissues. In both cases, lactate quantification was performed using the Promega Lactate-Glo™ Assay, a bioluminescent assay suitable for detecting lactate in either cell culture media or tissue samples. We have now clarified this distinction in the Methods section and corresponding figure legends to avoid confusion.*

- Figure 2A – I understand that you should not need to include every intermediate step in this figure panel, but I do think that one crucial step is missing, the conversion of lactate to lactyl-CoA prior to lysine acylation by ACSS2 (PMID: **39561764)**.

*Response: We agree that conversion of lactate to lactyl-CoA represents a critical intermediate step prior to lysine lactylation. Recent studies have proposed that enzymes such as ACSS2 may catalyze this process, although other candidates, including* *GTPSCS, have also been implicated (PMID: 39642882). However, the precise enzymatic mechanism responsible for lactyl-CoA generation remains under active investigation and may be context- or tissue-dependent. At present, the specific enzyme mediating lactyl-CoA formation in the retina has not been established. Because of this ongoing uncertainty, our schematic in Figure 2A was intentionally simplified to illustrate the conceptual link between lactate metabolism and histone lactylation without assigning a definitive enzymatic step.*

- Figure 3F and S2C – the level of colocalization with H3K18La and NRL seems stronger than it is portrayed in the text, and overlap between H3K27me3 also seems strong, if only because signal for H3K27me3 is everywhere. Could you use an image analysis tool (for example from imageJ) to better support or refute these claims related to colocalization on pages 10 and 11: “*Confocal microscopy using specific antibodies validates partial colocalization of H3K27Ac and H3K18La signals in the euchromatin region at the periphery of photoreceptor nuclei (Fig 3F). Little or no colocalization is detected between H3K18La and H3K27me3 (a mark associated with reduced gene expression) or NRL immunolabeling (S2C Fig). This suggests that H3K18La is enriched at euchromatic promoters in photoreceptors where it overlaps with H3K27Ac, while remaining distinct from the H3K27me3, a repressive mark.)”.*

*Response: We thank reviewer for this helpful suggestion. We have now performed the colocalization analysis and results are presented in the text within results (see page 15 of the revised maniuscript)*

- Figure 3J - I don’t quite understand this panel. It is supposed to represent H3K18La co-occupancy at promotors. Co-occupancy with what other factor? The y-axis says “intersection”, but I am not sure what thing that is intersecting is being counted. Some of the bar graphs are for only one tissue (retina, BMDM, ADIPO, and MB), others are for more than one, but there is no clear explanation of why particular tissue pairings are chosen to be shown here.

*Response: We agree. Using this UpSet plot, we wanted to illustrate the overlap of H3K18La promoter peaks across tissues. Specifically, we examined promoters marked by H3K18La in the retina and determined how many of these are also marked by H3K18La in other tissues which also produces and have been characterized in previous study (BMDM, adipocytes, myoblasts, etc.).*

*In this plot: The bar graph (top) represents the number of promoter peaks corresponding to each specific combination of tissues. The matrix below indicates which tissues are included in each combination. Single black dots represent promoter peaks unique to one tissue. Connected black dots represent promoter peaks shared among the indicated tissues. Thus, the y-axis quantifies the number of H3K18La-marked promoters corresponding to each specific intersection (i.e., unique or shared across tissues).*

*To improve clarity, we have revised the y-axis label from “Intersection” to “Number of promoter peaks” and expanded the figure legend to explicitly describe the UpSet plot representation and the meaning of single versus connected dots.*

- Figure 4F shows several different temporal patterns where transcript expression can increase before, during, or after increases in H3K18La. I am not a genomics expert, but does this not devalue the claim that H3K18La causes an increase in transcription? It seems to me that this panel shows evidence that H3K18La does not exert a strong level of control over transcription. Of course I understand that each of these genes will be subjected to transcriptional control by an abundance of other factors in addition to H3K18La, but there should

*Response: We are grateful for this insightful comment. The reviewer is correct, the heterogeneous temporal patterns shown in Figure 4F do not support a deterministic model where H3K18La directly drives transcription in all cases. Rather, our data suggest that H3K18La is associated with transcriptional changes in a context-dependent manner. As the reviewer notes, gene expression is regulated by multiple factors, including other histone modifications, transcription factors, chromatin accessibility, and developmental cues. In some cases, increases in H3K18La coincide with transcriptional activation, whereas in others the correlation is less temporally aligned such as here in RorB (Figure 4F). This variability is consistent with the idea that H3K18La may function as a permissive chromatin mark rather than a sole driver of transcriptional activation. Importantly, we did not intend to suggest that H3K18La alone is sufficient to induce transcription.*

*In response to this comment, we have revised the conclusion of the results, to avoid causal language and more clearly frame*  *that “**Together, these results suggest that H3K18La is gained at promoters already in a permissive chromatin state and is associated with developmental and neuronal programs involved in retinal maturation”.*

- Figure 5C – a label is missing on the right-most panel. I assume it is H3K27Ac.

*Response: We have now corrected the figure to include the appropriate label (H3K27Ac).*

- Figure 6G – a box around the different pathways (glycolysis vs. PPP vs. mitochondrial) will help to better indicate which gene is associated with which pathway.

*Response: We thank the reviewer for this suggestion. We have carried out an extensive revision to Figures 6 and 7 and have taken your suggestion, as requested.*

- Figures 6 and 7 - The manuscript could be simplified if the data in figures 6 and 7 were combined. The goal of these figures are to interrogate the epigenetic responses to increases (fig 6) and decreases (fig 7) in glucose metabolism. These modulations, place on a single scale, could yield a more comprehensive glycolytic dose-response effect.

*Response:* *We agree that presenting a single, comprehensive figure would be a powerful way to visualize the explant data. We have merged the two figures into a new, single, multi-panel Figure 6. Additionally, we have expanded supplementary figures. In this revised figure, the data are presented together to tell a complete story, but the quantitative plots remain as separate panels, each compared to its own control.*

- Figure 7G – This figure shows a rather poor correlation between changes in transcript levels and changes in H3K27Ac or H3K18La. Does an integrated measure of both H3K27Ac and H3K18La cut-and-tag datasets improve the ability of these histone marks to predict transcript levels?

*Response: Our 2-DG experiment indicates a modest, but noticeable, relationship between changes in lactylation and transcriptional output. As noted by the reviewer with the glucose explant experiment, this experiment was also performed at 48 hours post-2-DG treatment. We do not currently know the time dynamic relationship between 2-DG treatment and subsequent H3K18La changes and the ensuing transcriptional changes. At 48 hours we observe a modest change in transcription but a more robust change in H3K18La, perhaps at a later time-point we’d observe more robust transcriptional responses. Further study in the time dynamics of this system is an interesting avenue of future investigation. With regards to an integrated analysis, we observe that our most significant changes in transcription are associated with the most robust changes from both H3K18La and H3K27Ac suggesting a cooperative effect.*

- I am not a genomics expert, so I am not sure what information is gained from the PCA plots and the # of consensus peaks which isn’t supported by other elements of the manuscript. With the goal of simplifying the manuscript, I suggest moving these data to the supplemental text.

*Response: We have moved the PCA plot and number of consensus peaks for retinal explants experiment associated with Fig 6 in the supplementary information. However,* we respectfully submit that the PCA plots and consensus peak analyses are important components of the Figure 3. *The PCA plots demonstrating the high reproducibility between our biological replicates and in particular dynamics of H3K18La peak enrichment at P10. Similarly, the number of consensus peaks provides a quantitative measure of the magnitude of global chromatin alterations in our analyses.*

- Discussion – This is an opinion, but this section contains a lot of information typically destined for the introduction. I recommend removing the first ~8 lines of text in the first paragraph, and there are many examples of text which does not add to the section which should be focused on the interpretation of your findings rather than a re-introduction to the field.

*We sincerely appreciate the reviewer’s opinion. We believe that our manuscript is probably the first one to comprehensively capture and report the nexus of lactate-epigenome-gene regulation in the retina. This is a complex topic that deals with non-energy functions of lactate (the final metabolic product of glycolysis), and much more remains to be done. We have attempted to keep the Introduction concise and coherent and reserved it for primarily for introducing what is being done and why. In contrast, we have taken more liberty to expand and hypothesize. In the first paragraph of the Discussion, we have presented the big picture and how our findings have contributed. We then discuss the points that could not be included in Results to keep the data presentation concise and easier to comprehend. The examples are provided for explaining specific results. Though there is some redundancy, we believe it is helpful in explaining this complex topic to broader readership of PLoS Genetics.*

- Page 21 line 3. The sentence which begins on this line is totally unsupported by the reference (ref 51), which neither mentions the AARS1 gene or lactyl-CoA. A Pubmed search of “AARS1 and Lactyl-CoA” gives no results, though a google search supports the statement (PMID: **38653238**, 38512451). Perhaps this is an error with the reference manager (we’ve all dealt with these), but this means the authors should comb through and make sure all references are properly attributed.

*Response: We sincerely apologize for this error and thank the reviewer for carefully identifying the incorrect reference. This was indeed a reference manager oversight on our end. We have corrected the citation to include the appropriate references (**PMID: 38653238 and* *38512451), which support the discussion of AARS1 and lactyl-CoA mediated lactylation. In addition, we have carefully reviewed the entire manuscript to ensure that all references are correctly attributed and accurately support the corresponding statements.*

- Methods – there are catalog numbers missing from many items. To ensure reproducibility, please include these in the final submission of this manuscript.

We have added the catalogue

*Response: We apologize for the oversight. We have now updated the Methods section to include catalog numbers for all specialized reagents, including antibodies, enzymes, and kits, to ensure reproducibility. We have omitted catalog numbers for generic, common chemicals where the supplier is not a critical variable for the experimental results, in line with standard reporting practices.*

**Reviewer #2:**

This is an extensive analysis of histone lactoylation in mouse retinas. In carefully done and clearly reported studies the investigators show where histone lactoylation occurs and they show how it changes during development. They identify sites of lactoylation on histones and they quantify changes in gene expression that correlate with histone lactoylation. This report will be useful because it provides a foundation for future studies of this type of epigenetic modification and it identifies genes whose expression are likely to be affected by it.

*Response: We thank the reviewer for positive assessment of our study and appreciating the significance of our proposed model of lactate mediated regulation of histone lactylation for future studies.*

The authors should address the following questions:

1. In Fig. 2D there is a lot of H2K18 lactoylation in the inner retina even at P6, but Fig. 1B shows that there is not very much lactate being produced at P6 (based on extracellular acidification). Please discuss or speculate on where the lactate could come from to be a substrate for a level of inner retina lactylation at P6 that appears to be equal to or to be even more abundant that the amount of lactoylation in the outer retina at P2.

*Response: We thank the reviewer for raising this point. We agree that retinal layers can differ substantially in how tightly histone lactylation is linked to glycolysis. Based on our data, we have two observations: (i) in the photoreceptor layer, H3K18La changes linearly with change in ECAR, whereas (ii) in the inner retina, H3K18La is not as dynamically coupled to the glycolytic changes. Our interpretation is that the bulk ECAR measured from the entire retinal punch (Fig. 1B) is dominated by the metabolic activity of the outer retina (photoreceptors) known for high rates of aerobic glycolysis (the Warburg effect).* *The inner retina can have localized lactate production, particularly through the metabolic activity of Müller cells and which is sufficient to drive lactylation but does not proportionally contribute to the total tissue ECAR at P6. Also, the steady-state level of H3K18la is determined by both 'writer' (lactyltransferases) and 'eraser' (delactylases) enzymes. It is plausible that the inner retina at P6 has lower activity of the relevant histone delactylases, leading to an accumulation of the H3K18la mark even with a more modest supply of lactate*.

2. I think it is not accurate to state: "Together, these results demonstrate that glycolytic inhibition by 2-DG perturbs both lactoylation and acetylation landscapes in retinal explants, with partially overlapping yet distinct expression outcomes, providing an additional layer of regulation at a subset of stress and immune response-related genes". I feel that this might be too strong an interpretation for the reasons that the authors clearly describe in the preceding paragraph. In the experiments in Fig. 7, 2-DG is used to block glycolysis. That likely makes the cells very stressed, i.e. ATP levels in the cell probably drop substantially. (In fact it would be worthwhile and probably not too difficult, to quantify ATP under these conditions.) The amount of lactoyl CoA substrate for histone lactoylation likely is diminished. Low ATP levels also must affect transcription overall so it would make the findings from these experiments difficult to interpret unambiguously. Very low levels of nucleotide triphosphates must slow transcription and it may do that differently for every gene being transcribed (probably influenced by relative rates of transcription) independently of any specific stress responses.

*Response: We thank the reviewer for this thoughtful and important comment. We agree that 2-DG treatment can have pleiotropic effects, including reductions in ATP levels and broader metabolic stress, which could globally influence transcription independent of specific histone modifications. Our intention was not to imply that the observed transcriptional changes are solely or directly mediated by altered histone lactylation. To address this concern, we have revised the text and clarify that our findings* *demonstrate an association between glycolytic inhibition, altered H3K18La landscapes, and transcriptional changes, rather than establishing direct causality. We also note in the Discussion that broader metabolic effects of 2-DG may contribute to the observed gene expression patterns.*

3. Many of the experiments in this report are done by raising the concentration of glucose to produce more lactate. I think the authors are making the assumption that the only, or the primary, relevant effect of increased glycolytic flux is that more lactate is produced. But there also could be other effects of increasing glucose, like more NADH, more pentose phosphate pathway activity, more NADPH or more ATP. Wouldn’t it be possible to address this by measuring effects on lactoylation and gene expression by directly adding lactate instead of glucose? Also, would addition of pyruvate cause the same effects as adding glucose or adding lactate? What patterns of lactoylation would be produced if glucose was added at a high concentration but a glycolysis inhibitor like iodoacetate or oxamate was added?

*Response: The reviewer is entirely correct. We agree that increasing glucose availability can alter multiple metabolic parameters beyond lactate production, including cellular redox state (NADH/NAD⁺), PPP activity and NADPH generation, and ATP levels, any of which could influence chromatin lactylation and gene expression. Our rationale for using elevated glucose was to modulate lactate production through a physiologically relevant upstream driver of lactylation, i.e. glucose, but we agree that this approach alone does not isolate lactate-specific effects.*

*We agree that combining high glucose with glycolysis inhibition or LDH inhibition (e.g., oxamate) could help discriminate whether the observed lactylation changes require glycolytic flux and/or lactate production. We address this as limitation in our experimental strategies in the discussion and will consider this in the future studies.*

**Reviewer #3:** 

In this study, the authors focus on a potential connection between metabolism and gene expression via epigenetic control in the developing mouse retina. Specifically, they examine one histone modification, histone lactylation (H3K18-lactylation), that correlates with the increased glycolysis and lactate levels that they observe in the developing retina. This idea is intriguing because it would provide a really nice mechanism to connect metabolic changes with gene expression during retina development, and this could be particularly important for cells in the retina such as photoreceptors that are highly metabolically active. The authors provide some good correlative data supporting an increase in H3K18-lactylation that parallels glycolysis in the developing retina, but some of their conclusions are complicated by the fact that at least one acetylation mark – H3K27ac – seems to follow H3K18La very closely. This makes it difficult to separate out the impact of histone lactylation from acetylation. In addition, the transcriptional changes they observe don’t seem to correlate in one direction (positive or negative) with H3K18La, making it a bit hard to understand exactly what the role of this modification may be. Thus, some of the major conclusions of the paper as outlined in the title and abstract may be too strong based on the data provided. There are also some technical concerns regarding both the CUT&Tag analysis and antibody specificity that should be addressed because these data were critical for many of the conclusions of the study. Overall, this is an interesting study on an important area that could provide some insight into the connections between metabolism and the epigenome in the developing retina. We also want to commend the use of the retinal explant model that provided a very good approach to testing the role of glucose metabolism in altering chromatin marks and gene expression via direct manipulation of available glucose and 2-DG. Our specific major and minor concerns are outlined below.

*Response: We thank the reviewer for this thoughtful and comprehensive assessment of our study. We appreciate the recognition of the significance of the study and linking metabolic changes to epigenetic regulation in the developing retina.*

*We acknowledge the reviewer’s concerns regarding the overlap between H3K18La and H3K27Ac, the interpretation of transcriptional directionality, and the strength of some of our conclusions.* *Since H3K27Ac is a broad mark for active chromatin, it often colocalizes with H3K18La at promoters and enhancers, but our data reveal distinctions between H3K27Ac and H3K18La as well. We noted clear differences in their genomic distribution and their dynamic response to high glucose and glycolysis inhibition in retinal explants. We also find distinct transcription factor motif binding sites on these two histone marks.*

*In line with previous studies, our analysis indicates a complementary role for H3K18La, which acts in concert with H3K27Ac at cis-regulatory regions to mediate the expression of genes essential for cell identity. As we have shown, the enrichment of H3K18La on pre-existing permissive chromatin at P10 serves to increase the expression of genes important for photoreceptor function. Furthermore, it is established that p300 catalyzes multiple acylations, and it is hypothesized that the specificity of these modifications is determined by substrate abundance in the nucleus. Our study provides an experimental framework to test this hypothesis.*

*In response, we have revised the conclusion in the Results, Abstract and Discussion to moderate statements that may have been overstated and to more clearly interpret our findings as correlative rather than causative.*

Major concerns:

1. Apart from the mass spectrometry data in Fig. 2, most assays (WB, IHC, CUT&Tag) that assess H3K18La in this study used the same antibody: anti-H3K18La (Rabbit, PTM1406RM, PTM Bio). We recommend that the authors carefully check the specificity of this antibody to confirm that it doesn’t cross-react with H3K18ac or H3K27ac, especially when H3K18La and H3K27ac are found in many shared regions. Some discussion of this point should be included and/or specificity controls if appropriate.

*Response: We appreciate reviewer's critical evaluation of the anti-H3K18La antibody's specificity, which is central to our conclusions. The selected H3K18La, PTM1406RM, is extensively characterized in the original paper describing histone lactylation (Zhang et al., Nature, 2019 ref#7). Specifically, the authors performed peptide dot blot assays (see Extended Data Fig. 1c in their paper) where the antibody showed a strong and specific signal for the H3K18La peptide, but no cross-reactivity with peptides containing H3K18Ac, H3K27Ac modifications. To address the reviewer's point directly in our manuscript, we have now added the following sentence to the Methods section: 'The specificity of the anti-H3K18La antibody (PTM1406RM) was previously validated by peptide dot blot, confirming no cross-reactivity with H3K18Ac or H3K27Ac (Zhang et al., Nature, 2019)’. We believe this established validation, now mentioned in our text, sufficiently addresses this important concern.*

2. Since H3K18La is deposited by histone acetyltransferase p300, which also acetylates H3K18ac and H3K27ac, it is possible that the observed H3K18La changes reflect a byproduct of p300-mediated acetylation activity. Therefore, inclusion of appropriate controls would be important to distinguish these possibilities. Specifically, in Fig.2D, in addition to pan-lactyl and H3K18La, we recommend assessing H3K18ac and/or H3K27ac, and pan-acetylation changes at least at two representative stages. In addition, if H3K27ac and H3K18ac turn out to change during development, acetyl-CoA levels should be measured as well.

*Response:* *We thank the reviewer for this important and mechanistically insightful comment. We agree that p300 can catalyze multiple acetyl and non-acetyl modifications, including H3K18Ac, H3K27Ac, and H3K18La, and that changes in H3K18La could, in principle, reflect broader alterations in acetyltransferase activity of p300. While p300 mediates both lactylation and acetylation, they are not always strictly coupled. Studies have shown that in some contexts, such as certain* *cardiovascular disease models, H3K18La levels can change significantly while histone acetylation levels remain largely unchanged (PMID: 39205384). Furthermore, other enzymes, such as AARS1, have been identified as having lactate transferase activity, suggesting that p300 may not be the only enzyme regulating histone lactylation. However, this is an evolving area of research and need further validations in the physiological contexts.*

*Our goal in this study was to characterize the dynamics of histone lactylation in the developing retina, which exhibits changes in glycolysis, rather than to assign enzymatic specificity or to dissect the relative contributions of acetyl-CoA versus lactyl-CoA availability. We therefore interpret the observed changes in H3K18La primarily as reflecting shifts in glucose metabolism, without concluding that these changes can be attributed to p300 activity.*

*We agree that systematic comparison with acetylation marks (e.g., H3K18Ac, H3K27Ac) and direct measurements of acetyl-CoA would further clarify the relationship between acetylation and lactylation during retinal development. We now explicitly acknowledge this possibility in the revised Discussion and frame it as an important direction for future mechanistic studies.*

3. In Fig.2B, the mass spectrometry data are important for supporting H3K18La as a relevant target, but the current analysis lacks quantification. The claim that “lysine lactylation is abundant across histones” is not supported by a figure. Moreover, it is unclear whether H3K18La is more abundant than other histone modifications and why it was selected for follow-up. Some quantification or discussion of the relative abundance of this particular mark relative to other histone marks (eg acetylation) would be helpful here.

*Response: We agree that our mass spectrometry analysis was not designed to provide quantitative measurements of histone lactylation abundance. Rather, our goal was to perform site identification in retinal tissue and determine whether established lactylation sites, including H3K18, could be detected in vivo.* *We have revised the manuscript to clarify that the MS data are qualitative and support the presence of multiple histone lactylation sites, rather than their relative abundance.*

*We also agree that our original phrasing suggesting that lysine lactylation is “abundant” across histones was not sufficiently supported by the data. This statement has been revised to avoid implying quantitative conclusions.*

*H3K18La was selected for further analysis for two primary reasons:
(i) it is one of the most well-characterized lactylation sites in the literature, and
(ii) in our western blot analyses, H3K18La exhibited more dynamic changes compared to other tested sites (e.g., H4K12La). Importantly, we do not conclude that H3K18La is more abundant than other lactylation or acetylation marks, and we now clarify this distinction in the revised text.*

4. We are a little concerned about the CUT&Tag data quality as no negative control (e.g., anti-IgG) was included. A negative control is important for reliable peak calling and for meaningful comparisons of signal across conditions since CUT&Tag, like other chromatin profiling techniques, often has some non-specific background. We therefore recommend that the authors either repeat at least one CUT&Tag experiment at P28 with the inclusion of an IgG control, or incorporate high-quality, stage-matched public IgG CUT&Tag data from mouse retina for all peak-based analyses. Considering the authors’ focus on the co-localization of H3K27ac and H3K18La, accounting for non-specific binding is necessary to reduce potential false-positives. In addition, we suggest including the ENCODE blacklist during peak calling. Based on the bigwigs shown for some of the P4 retinas, we are not sure that the peaks identified represent any real signal at this stage – background controls may help here as well.

*Response: We thank the reviewer for this critical feedback regarding the necessity of a negative control for our CUT&Tag analysis. We agree completely that a robust background control is essential for reliable peak calling and for potential false positives, particularly for our co-localization analysis. Regarding the blacklist: We apologize for not stating this clearly in our original methods. We did, in fact, use the ENCODE mm10 blacklist (v2) during our initial peak calling. We have now clarified this in the revised Methods section to ensure our pipeline is transparent. Regarding the IgG control: This is the more crucial point. Our protocol was based on the manufacturer's (Active Motif) recommendation, which suggested an IgG control may not be necessary and does not add any additional value as the pA-Tn5 was shown to be specific (*[*https://www.activemotif.com/documents/ActiveMotif_Cut&Tag_eBook.pdf#:~:text=A.%20The%20purpose%20of%20including,the%20antibody%20is%20located%2Fenriched*](https://www.activemotif.com/documents/ActiveMotif_Cut&Tag_eBook.pdf#:~:text=A.%20The%20purpose%20of%20including,the%20antibody%20is%20located%2Fenriched)*.)*

*Nevertheless, we performed pilot experiments including both IgG and no-antibody controls in several runs. However, the resulting libraries were highly inconsistent and frequently yielded insufficient signal to generate reproducible peaks or to serve as a reliable normalization reference for the antibody of interest. These findings are consistent with reports that IgG controls in CUT&Tag often produce very low-complexity libraries that are not suitable for quantitative normalization.*

5. In connecting the increase of H3K18La to transcriptomic data in Fig 4C, we are concerned that the analysis focuses exclusively on positively correlated genes, while almost an equal number of genes show negative correlations with increased H3K18La. In addition, the conclusion that H3K18La activates transcription in a permissive chromatin context appears to be based solely on the co-occurrence of H3K18La and increased gene expression at a small number of genes, which is insufficient to support a general activating role.

*Response: We thank the reviewer for this thoughtful comment. We agree that our results emphasized positively correlated genes and did not sufficiently discuss the negatively correlated subset. Consistent with previous work (e.g., Galle et al. ref # 38), which demonstrated that H3K18La preferentially marks tissue-specific enhancers and regulates specific gene programs rather than acting as a global transcriptional activator, our data similarly suggest a context-dependent role. Similarly in the original paper from Zhang et al they reported that H3K18La marks a subset of highly active promoters in hypoxic macrophages rather than uniformly activating transcription across the genome.*

*Our analysis of the negatively correlated genes reveals they are enriched for pathways like* *cytoplasmic translation, mitotic cytokinesis etc which are constitutive biological processes required for the basic maintenance of cell function. We posit that in these cases, H3K18la may represent a poised or "primed" state, where the chromatin is accessible, but secondary regulatory layers (such as H3K27me3 binding or H3K9me3 binding) dictate the final transcriptomic output. Instead, our findings support a model in which H3K18La functions in a context-specific manner, potentially contributing to transcriptional regulation within a permissive chromatin environment rather than acting as a primary instructive activation mark. Importantly, we have revised the conclusion of the results to “Together, these results suggest that H3K18La is gained at promoters already in a permissive chromatin state and is associated with developmental and neuronal programs involved in retinal maturation”*.

Minor concerns:

6. In Fig. 2C-D, the increase in H3K18La appears more pronounced in the ONL by IHC than by western blotting. Since IHC shows cell type-specific changes in H3K18La, whereas the WB samples represent mixed cell populations, we recommend quantifying IHC data rather than the WB results. Inclusion of some other histone marks (eg H3K27ac, H3K18ac) in these IHC data could also be helpful to show if this increase is unique to lactylation or not.

*Response:* *We agree that there are apparent differences between our IHC and western blot results, but these likely reflect the distinct nature and resolution of the two techniques. Here, IHC allows visualization of cell type–specific changes, such as those occurring in the ONL, whereas western blot data measures global histone protein levels from mixed retinal cell populations. Importantly, in the mammalian retina, photoreceptors constitute the predominant cell population and represent the majority of nuclei within the retina. Therefore, whole-retina western blot analysis is expected to largely reflect changes occurring in photoreceptors. We believe this explains why the western blot data remain consistent with the overall increase observed in the ONL.*

*Regarding the inclusion of additional histone marks (e.g., H3K27Ac or H3K18Ac), we agree that such analyses would be valuable to further dissect the specificity of the observed changes. However, as the primary aim of this study was to characterize the dynamics of histone lactylation in the developing retina, we have focused our analysis on H3K18La. We have acknowledged this as a limitation and noted that future studies should examine additional histone modifications (H3K18Ac, H3K27Ac). Of course, it will be important to determine whether the observed increase is unique to lactylation (H3K18La).*

7. In Fig. 2C, only H3K18La increased with development whereas pan- and H4K12La do not significantly change. Considering the proposed model, it is unclear as to why H3K18La in particular is increasing. Is there a reason why this appears to be specific to H3K18La?

*Response: We thank the reviewer for this insightful comment. Histone lactylation has been linked to glycolysis and also to availability of lactate, but another major regulator of these marks is the enzyme p300 (more predominantly) that use lactate as a substrate and catalyze lactylation. Kinetic studies show that p300 has a specificity for H3K18 site, that is significantly higher than for other residues like H3K14 or H4K12 as shown previously (PMID:* [*25325435*](https://pubmed.ncbi.nlm.nih.gov/25325435/)*) for acetylation. Moreover, the original report from Zhang et al 2019 reference # 7 in the manuscript, describing histone lactylation showed that H3K18 is particularly responsive to metabolic flux, further supporting our focus on this residue.*

8. In Fig. 3C, the authors should check H3K18La levels on peaks rather than (or in addition to) on genes (tss) in the metaplots, which might provide more consistent results. Fig. 3B appears to be redundant. There is no y axis label in Fig. 3C.

*Response: We thank the reviewer for these valuable suggestions to improve the clarity and rigor of our analysis. The reviewer's suggestion to analyze H3K18La levels on peaks was completed and showed consistent binding on the called peaks. However, the dynamics of the peak binding during development, as seen in Fig 3C, is lost since we interrogate the called peaks as opposed to all the genes.*

*Clarification on Figure 3B: We appreciate the feedback on the potential redundancy of Figure 3B. We respectfully clarify the non-redundant purpose of each panel. Figure 3B provides the genomic location of the peaks, showing the distribution across promoters, enhancers, gene bodies, etc. In contrast, Figure 3C shows the signal intensity at those peaks, illustrating the quantitative changes across development. We believe presenting both the qualitative assessment of the identified peaks (Figure 3B) and the quantitative dynamics in Figure 3C provides a more complete biological picture of H3K18La mark. Missing Y-axis Label: We apologize for this oversight. The y-axis label for Figure 3C has been added in the revised manuscript.*

9. Fig. 3E, to compare with colocalization of H3K18La and H3K27ac, we recommend first presenting the overall number of overlapping peaks or genes before highlighting region-specific overlaps.

*Response: We thank the reviewer for this helpful suggestion. While we agree that reporting global overlap can be informative in certain contexts, our primary objective in Fig. 3E was to assess region-specific co-localization patterns rather than overall genomic overlap. Because H3K27Ac is broadly distributed across active promoters and enhancers, reporting total overlap across the genome would largely reflect the global abundance of H3K27Ac peaks rather than provide mechanistic insight into locus-specific relationships with H3K18La.*

*To avoid potentially misleading interpretations driven by differences in peak abundance and genomic coverage, we focused our analysis on region-specific overlaps, which more directly address the biological question of whether H3K18La preferentially co-localizes with H3K27Ac at defined regulatory elements. We have clarified this rationale in the revised manuscript.*

10. The reproducibility of Fig.4’s P10 data for H3K18La CUT&Tag peaks is concerning. The authors address this by stating that the variability may be a result of active differentiation in these tissues. However, the RNA-seq data does not support the variability of replicates at the gene expression level. With only two replicates of great variability, the P10 H3K18La CUT&Tag is difficult to interpret.

*Response: We thank the reviewer for raising this important concern regarding the reproducibility of the P10 H3K18La CUT&Tag data. We agree that variability between the biological replicates at P10 is greater in CUT&Tag and we acknowledge that the limited number of replicates constrains the strength of conclusions that can be drawn from this dataset. Importantly, chromatin profiling and transcriptomic profiling capture distinct regulatory layers. While RNA-seq reflects steady-state mRNA abundance, CUT&Tag measures chromatin occupancy, which can be more dynamic and cell-state dependent.* *P10 represents a period of substantial cellular and chromatin remodeling in the developing mouse retina. H3K18La is only one factor driving gene expression of many, however there is huge transition in H3K18La enrichment from diffuse peak binding to robust peak binding at P10 and it illustrates the nature of the dynamics rather than technical noise alone.*

*Nevertheless, we agree that the variability warrants cautious interpretation. Therefore, we have tempered our conclusions regarding the P10 dataset and now present these findings as indicative of dynamic remodeling rather than definitive quantitative differences.*

11. In Fig. 5, what is the rationale for measuring H3K27ac levels rather than pan-acetyl or H3K18ac upon glucose or 2-DG treatment?

*Response: We thank the reviewer for this insightful comment. We chose to measure H3K27Ac because previous studies have shown that H3K27Ac marks active chromatin regions and can functionally overlap with histone lactylation marks, including H3K18La, particularly in the regulation of transcriptionally active genes. (Galle et al, ref # 38) Therefore, we used H3K27Ac as a representative acetylation mark associated with transcriptional activation. However, we fully acknowledge the reviewer’s point that assessing H3K18Ac specifically or using a pan-acetyl antibody would provide a more direct comparison to H3K18La and better distinguish between acetylation and lactylation specific effects under glucose or 2-DG treatment. We agree that this would strengthen the mechanistic interpretation. To address this concern, we have clarified our rationale in the revised manuscript as “We chose H3K27Ac as as a representative acetylation mark associated with transcriptional activation” in Figure 5 and acknowledged this limitation in the Discussion section as “Moreover, our analysis focused on H3K27Ac as a control marker for transcriptionally active chromatin in response to metabolic shifts, though we acknowledge that a direct comparison with H3K18Ac would resolve the site-specific interplay between lactylation and acetylation at this residue”.*

12. For the text relating to Fig. 6, stating that “Lactate promotes enrichment of H3K18La” is not entirely supported by the data. Lactate availability appears to correlate with H3K18La levels. However, only glucose was supplemented. Therefore, it cannot be determined whether lactate does indeed promote enrichment of H3K18La in these cells or another process impacted by glucose availability.

*Response: We thank the reviewer for this thoughtful comment. We agree that our original test overstated the conclusion. In the experiments shown in Fig. 6, glucose levels but not lactate was supplemented, and thus we cannot directly conclude that lactate itself promotes enrichment of H3K18La. Rather, our data demonstrate a correlation between glucose availability and H3K18La levels. To address this concern, we have revised the heading to state that “*Glucose availability and glycolytic inhibition (2-DG) differentially affect H3K18La dynamics”*. This is now revised Figure 6 merged with previous Figure # 7.*

13. Fig. 6B appears to be unnecessary. In Fig. 6C and 6E, the increased H3K27ac signal in the metaplot seems to conflict with the conclusion in Fig. 5C that H3K27ac remains unchanged under high-glucose treatment. We suggest clarifying this apparent discrepancy.

*Response: We thank reviewer for these helpful comments (Fig 6 and 7 are now combined in the revised manuscript). We respectfully submit that Fig. 6B is necessary. This panel provides complementary information to Fig. 6C, which is now supplementary Fig. S3B. Specifically, Fig. 6B illustrates the genomic distribution and overall landscape of H3K18La associated regions, whereas Fig. S3B focus on quantitative profiles at defined genomic features. Regarding the apparent discrepancy between the H3K27Ac western blot data and the CUT&Tag analyses, we appreciate the opportunity to clarify this point. In Fig. 5C, the western blot analysis did show a trend in increase in H3K27Ac levels under high-glucose treatment; however, this increase does not reach statistical significance. In contrast, the metaplot analyses from CUT&Tag, and Fig. 6B, illustrate H3K27Ac signal at specific genomic loci. The observed increase here reflects localized enrichment at defined regulatory regions rather than a genome-wide increase. Thus, while global H3K27Ac levels remain statistically unchanged, locus-specific redistribution or enrichment can still occur.*

14. Fig. 7 could be combined with Fig. 6 for better comparison. In Fig. 7C, the scale of the metaplot is not the same for control and 2-DG treatment. We would also like to see a metaplot for H3K27ac after 2-DG treatment. Similar to Fig. 6C, H3K18La levels show little change after 2-DG, which is inconsistent with the WB result in Fig. 5D.

*Response: We thank the reviewer for these constructive suggestions. We have merged the two figures into a new, single, multi-panel Figure 6. In this revised figure, the data are presented side-by-side to tell a complete story, but the quantitative plots remain as separate panels, each normalized to its own internal control. The difference in the y-axis scale in Fig. 7C (now this supplementary figure S4B) was the result of independent sample heatmap generation. We have revised Fig S4B to include H3K27Ac, as suggested, and have generated the heatmaps using the same overall scale.*

*Further Western blot analysis (Fig. 5D) measures global protein abundance of H3K18La across the entire chromatin pool. In contrast, CUT&Tag assesses genomic localization and enrichment at specific regions. Therefore, a global change detected by Western blot does not necessarily translate into proportional changes at the subset of loci represented in metaplot analysis for CUT&Tag*.

15. Fig. 8 (motif enrichment) should be in the supplemental – this analysis doesn’t have a strong connection to the rest of the manuscript and there are no follow-up studies on the transcription factors identified.

*Response: We thank the reviewer for this thoughtful comment. We respectfully prefer to retain this analysis as a main figure (now Fig. 7), as it provides important mechanistic context for the preceding results. The motif enrichment analysis represents a first step toward identifying candidate transcription factors that may mediate the context-specific effects of H3K18La on gene expression. Given that transcription factors play a central role in coordinating histone mark deposition as well as linking these marks to transcriptional regulation, this analysis helps integrate our chromatin and transcriptional findings into a coherent regulatory framework. While functional validation of the identified transcription factors is beyond the scope of the present study, this analysis generates testable hypotheses and establishes a mechanistic basis that will guide future investigations.*
